# ONE SHOT - single shot radiotherapy for localized prostate cancer: study protocol of a single arm, multicenter phase I/II trial

**DOI:** 10.1186/s13014-018-1112-0

**Published:** 2018-09-04

**Authors:** Thomas Zilli, Marta Scorsetti, Daniel Zwahlen, Ciro Franzese, Robert Förster, Niccolò Giaj-Levra, Nikolaos Koustouvelis, Aurelie Bertaut, Michel Zimmermann, Giuseppe Roberto D’Agostino, Filippo Alongi, Matthias Guckenberger, Raymond Miralbell

**Affiliations:** 1 Radiation Oncology, Geneva University Hospital, CH-1211, 14 Geneva, Switzerland; 2 Faculty of Medicine, Geneva University, Geneva, Switzerland; 3 Radiation Oncology, Humanitas University, Rozzano, Milan, Italy; 4 Radiation Oncology, Humanitas Research Hospital and Cancer Center, Rozzano, Milan, Italy; 5 Radiation Oncology, Kantonsspital Graubünden, Chur, Switzerland; 6 Radiation Oncology, University Hospital Zürich, Zürich, Switzerland; 7 Radiation Oncology, Sacro Cuore Don-Calabria, Negrar, Italy; 8 Methodology and biostatistic unit, Centre Georges François Leclerc, Dijon, France; 9 Faculty of Medecine, University of Brescia, Brescia, Italy

**Keywords:** Prostate cancer, Stereotactic body radiotherapy, Urethra-sparing, Single fraction, Quality of life, Electromagnetic transponders

## Abstract

**Background:**

Stereotactic body radiotherapy (SBRT) is an emerging treatment alternative for patients with localized prostate cancer. Promising results in terms of disease control and toxicity have been reported with 4 to 5 SBRT fractions. However, question of how far can the number of fractions with SBRT be reduced is a challenging research matter. As already explored by some authors in the context of brachytherapy, monotherapy appears to be feasible with an acceptable toxicity profile and a promising outcome. The aim of this multicenter phase I/II prospective trialis to demonstrate early evidence of safety and efficacy of a single-fraction SBRT approach for the treatment of localized disease.

**Methods:**

Patients with low- and intermediate-risk localized prostate cancer without significant tumor in the transitional zone will be treated with a single SBRT fraction of 19 Gy to the whole prostate gland with urethra-sparing (17 Gy). Intrafractional motion will be monitored with intraprostatic electromagnetic transponders. The primary endpoint of the phase I part of the study will be safety as assessed by CTCAE 4.03 grading scale, while biochemical relapse-free survival will be the endpoint for the phase II. The secondary endpoints include acute and late toxicity, quality of life, progression-free survival, and prostate-cancer specific survival.

**Discussion:**

This is the first multicenter phase I/II trial assessing the efficacy and safety of a single-dose SBRT treatment for patients with localized prostate cancer. If positive, results of ONE SHOT may help to design subsequent phase III trials exploring the role of SBRT monotherapy in the exclusive radiotherapy treatment of localized disease.

**Trial registration:**

Clinicaltrials.gov identifier: NCT03294889; Registered 27 September 2017.

## Background

Total dose and dose per fraction play an important role in the curative treatment of prostate cancer with radiotherapy (RT). Modern image-guided external RT allows safe dose escalation for prostate cancer. Doses above 74 Gy with conventional fractionation (2 Gy/day) have been shown to be beneficial. There are strong radiobiological considerations that suggest that treatment with a small number of large fractions (hypofractionation) may increase the therapeutic ratio of RT for prostate cancer.

The “α/β” ratio (i.e., cell sensitivity/cell repair) is a key parameter of the cell-survival linear-quadratic (LQ) model that purportedly defines the sensitivity of each tissue (healthy or tumoral) to changes in treatment fractionation. Prostate cancer cells may have a low α/β ratio (i.e.,~ 1.5 Gy), lower than that of most tumors or even of the late-responding normal tissues surrounding the tumor, the rectum and the bladder (α/β ratio 3–5 Gy) [1]. Thus, hypofractionation may increase the tumor cell killing effect with relatively less toxic effect on the surrounding late responding normal tissues compared to conventional fractionation. Several authors have reported their respective experiences with moderate hypofractionation (i.e., dose per fraction 2.5 Gy to 4 Gy) for prostate cancer confirming the equivalence in terms of disease control and tolerance compared to standard fractionated treatments [2–5].

Bio-mathematical models using large patient data sets have focused on external beam RT schedules using less than 4 Gy fractions. It deserves to be acknowledged that the LQ model may not be predictive of cell survival and isoeffects with doses per fraction above 3-4Gy. Indeed, the predictions of the LQ model at very high dose/fraction (extreme hypofractionation) may be somehow “ambivalent”, either overestimated (less repair of sublethal damage thus a lower value for “β”) or underestimated (increased indirect cell-death secondary to intravascular endothelial damage). Staring from this background, clinical research on extreme hypofractionation started some 15 years ago when stereotactic body RT (SBRT) technology appeared as a treatment option against localized prostate cancer competing with high-dose rate brachytherapy (HDR-BT).

Indeed, preliminary results on extreme hypofractionation with SBRT have been reported during the last few years mainly for low-risk patients in single-institution [6–8] or multi-institutional [9] series. Most frequently, 5 fractions of 7.25 Gy have been delivered for a total equivalent dose to the tumor of 90 Gy in 2 Gy/fraction (LQ model) and a success rate of > 90%, 5-year biochemical relapse-free (bRFS) survival rates [10]. Data published so far for prostate SBRT have shown, although with a limited follow-up, late grade 3 toxicity rates for rectal and genitourinary (GU) toxicity lying within a ≤ 3% range [8, 11, 12]. Although, caution is recommended to avoid passing a too optimistic message regarding the potential biological benefits of extreme hypofractionation for prostate cancer (i.e., more cure with less side effects), financial and logistical advantages can undoubtedly be foreseen. In fact, a drop from 40 or more treatments to 5 or less sessions may reduce the cost of external beam RT, will increase the availability of treatment slots in otherwise busy departments (important issue in countries with limited resources), and significantly improve patient’s convenience.

The question of how far can the number of fractions with SBRT be reduced is an exciting research matter with an undoubtful goal, face the challenge of assessing the potential for cure of prostate cancer patients with a single and unique fraction of high dose irradiation similar to what is already undertaken with radiosurgery against brain, lung, and liver targets. Such type of effort has already been attempted with HDR-BT. Recently, Prada et al. [13] reported a relatively disappointing 66% 6-year bRFS survival though a very good tolerance after a single interstitial application of 19 Gy to the prostate. Reasons for this suboptimal result may be related, for instance, to a suboptimal dose-distribution with HDR-BT compared to SBRT. Hoskin et al. have recently published the results on 49 patients treated with a single fraction of HDR-BT to 19/20 Gy [14]. With 49 months median follow-up, the 4-year estimates of grade-3 GU and gastrointestinal (GI) toxicity was 2% and 0%, respectively, with no grade 4 events, while the 4-year bRFS was 94%. Similarly, Morton et al. confirmed with a median follow-up time of 20 months the good tolerance of a single 19 Gy single HDR-BT fraction in terms of toxicity and impact on quality of life (QoL) [15] and Krauss et al. reported a 3-year bRFS rate of 93% [16], similar to results observed by Hoskin et al. In conclusion, for the authors one single fraction HDR-BT is feasible, weakly toxic, and with promising preliminary results.

Although, the effects are mostly encouraging, only controlled studies are able to properly address the impact of single fraction SBRT in prostate cancer outcomes. Therefore, a phase I/II approach will be a properly controlled study to demonstrate early evidence of safety and efficacy of the intervention and substantiate further the role of a single-fraction SBRT approach. If positive, the results of ONE SHOT will help determine the design of subsequent phase III trials in the exclusive RT setting for the treatment of localized disease.

## Methods/Design

This study was approved by the Ethics committee of the Geneva University Hospital (2017–01236) and is registered on clinicaltrials.gov (NCT03294889). This is a phase I/II, prospective, multicenter, single-arm study.

### Phase I, safety evaluation

The main objective of the phase I trial is to determine if a single fraction SBRT, with a dose of 19 Gy, is safe and well tolerated by assessing the occurrence of Grade ≥ 3 acute adverse events (AE) during the first 3 months.

### Phase II, efficacy evaluation

The main objective of the phase II trial is to determine if a single fraction SBRT, with a dose of 19 Gy, is an effective treatment option by assessing bRFS at 3 years.

#### Objectives

##### Primary endpoint of the phase I trial

Grade ≥ 3GU and GI acute AE will be assessed during the first three months following the single fraction treatment. AEs will be assessed according to National Cancer Institute Common Terminology Criteria for Adverse Events (NCI CTCAE) v4.03. Special attention will be given to diarrhea, fecal incontinence, proctitis, rectal hemorrhage, rectal pain, hematuria, urinary frequency, urinary urgency, urinary retention, urinary incontinence, and cystitis non-infective. Persistent AE will be defined as any persistent toxicity during the first 3 months post-treatment (follow-up milestones: 1 week, 6 weeks, and 12 weeks).

##### Primary endpoint of the phase II trial

bRFS, from time of inclusion until biochemical progression (according to recommendations of the Radiation Therapy Oncology Group - American Society for Radiation Oncology (RTOG-ASTRO), the Phoenix Consensus Conference recommendations) [17]:

- A rise by 2 ng/ml or more above the nadir prostate specific antigen (PSA) confirmed by a second observation taken 3–4 weeks later, where the nadir PSA is defined as the lowest PSA value observed on study.

##### Secondary endpoints of the phase II trial

- Acute AE (during the first 3 months follow-up) according to CTCAE v4.03
- Late AE (after 3 months follow-up) according to CTCAE v4.03
- Progression free survival (PFS) defined as time from inclusion until one of the following events, whichever comes first:
- Biochemical progression
- Clinical progression defined as either local or regional recurrence of the disease or the appearance of distant metastases
- Death from any cause
- Start of another line of systemic anti-neoplastic therapy
- Clinical progression free-survival
- Local progression free-survival
- Time to further anti-cancer therapy defined as the time from inclusion to the start of any type of salvage treatment.
- Prostate cancer-specific survival (PCSS)
- Overall survival (OS)
- QoL evaluated using EPIC 26 (Expanded prostate cancer index composite) [18], IPSS (International Prostate Symptoms Score) [19], and IIEF-5 (International Index of Erectile Function) [20]

#### Inclusion criteria

- Written informed consent according to ICH/GCP regulations before registration and prior to any trial specific procedures
- Histologically confirmed adenocarcinoma of the prostate without small cell features
- Tumor clinical stage cT1c-2c, pN0 or cN0, M0, according to UICC TNM 2009
- Magnetic Resonance Imaging (MRI) staging must confirm American Joint Committee on Cancer (AJCC) stage T1, T2a, T2b, or T2c
- Gleason score at biopsy 3 + 3 or 3 + 4 (World Health Organization, WHO 2016 Grade Groups 1, 2)
- PSA ≤15 ng/ml
- Age 18–85 years at time of registration
- WHO performance status 0–1
- IPSS ≤10 (alpha blockers allowed)
- MRI-based volume estimation of prostate gland ≤70 cc
- Patient agrees not to father a child during trial treatment and during 6 months thereafter

#### Exclusion criteria

- Evidence of T3a, T3b, or T4 disease as assessed by MRI
- Positive lymph-nodes or metastatic disease from prostate cancer on imaging studies.
- Significant tumor on the transitional zone as assessed by MRI
- Previous or ongoing androgen deprivation therapy
- Impossibility to implant electromagnetic transponders into the prostate
- History of hematologic or primary solid tumor malignancy, unless in remission for at least 3 years from registration with the exception of curatively treated localized non-melanoma skin cancer
- Prior pelvic RT
- Previous surgery for prostate cancer
- Previous transurethral resection of the prostate (< 12 weeks before registration)
- Hip prosthesis
- Severe or active co-morbidity likely to impact on the advisability of SBRT
- Any other serious underlying medical, psychiatric, psychological, familial, or geographical condition, which in the judgment of the investigator may interfere with the planned staging, treatment and follow-up, affect patient compliance, or place the patient at high risk from treatment-related complications

#### Intervention

In this trial, patients with localized prostate cancer are registered to receive the following target SBRT dose:

- 19 Gy in a single fraction to the whole prostate gland ± proximal seminal vesicles
- 17 Gy in a single fraction to the urethra planning-risk volume (PRV)

Before the SBRT treatment, all patients will be implanted transrectally or transperineally under ultrasound guidance with three electromagnetic transponders, the *Calypso*® beacons (*Varian Medical Systems, Palo Alto, CA*). Patients will be simulated a minimum of 4 days after the implant and treated with an empty rectum and full bladder. To help with the contouring of the urethra a 12 *French Foley* non-radiopaque catheter will be inserted before the CT simulation and before irradiation. Rigid or deformable co-registration with multi-parametric MRI is to be used for contouring purposes.

The Clinical Tumor Volume (CTV) is defined as the prostate +/− the proximal 2/3 of the seminal vesicles (SV) based on the risk of SV involvement as determined by the *Roach* score using a cutoff threshold of 15% [21]. The planning target volume (PTV) is defined as the CTV plus 5 mm margins in all directions except for a 3 mm margin posteriorly towards the rectal wall. The urethra PRV is defined on CT images by contouring a 12 *French Foley* catheter with a 2 mm isotropic rim expansion. Organs at risk (OAR) are contoured according to RTOG guidelines [22] and include the bladder wall and the rectal wall (both defined as a 5-mm internal margin created from the external surface), the penile bulb [23], and the proximal femurs. All patients will be treated with megavoltage beams from a *TrueBeam®* linear accelerator (*Varian Medical Systems, Palo Alto, CA*) with nominal photon energies ≥6 MV, 6 degrees of freedom couch, and a *Calypso*® localization system. The irradiation technique is *RapidArc*® (*Varian Medical Systems, Palo Alto, CA*), a rotational technique using a flattening filter-free (FFF) modality with patients treated in an isocentric setting.

##### Dose prescription, recording and reporting

**Fig. 1:**
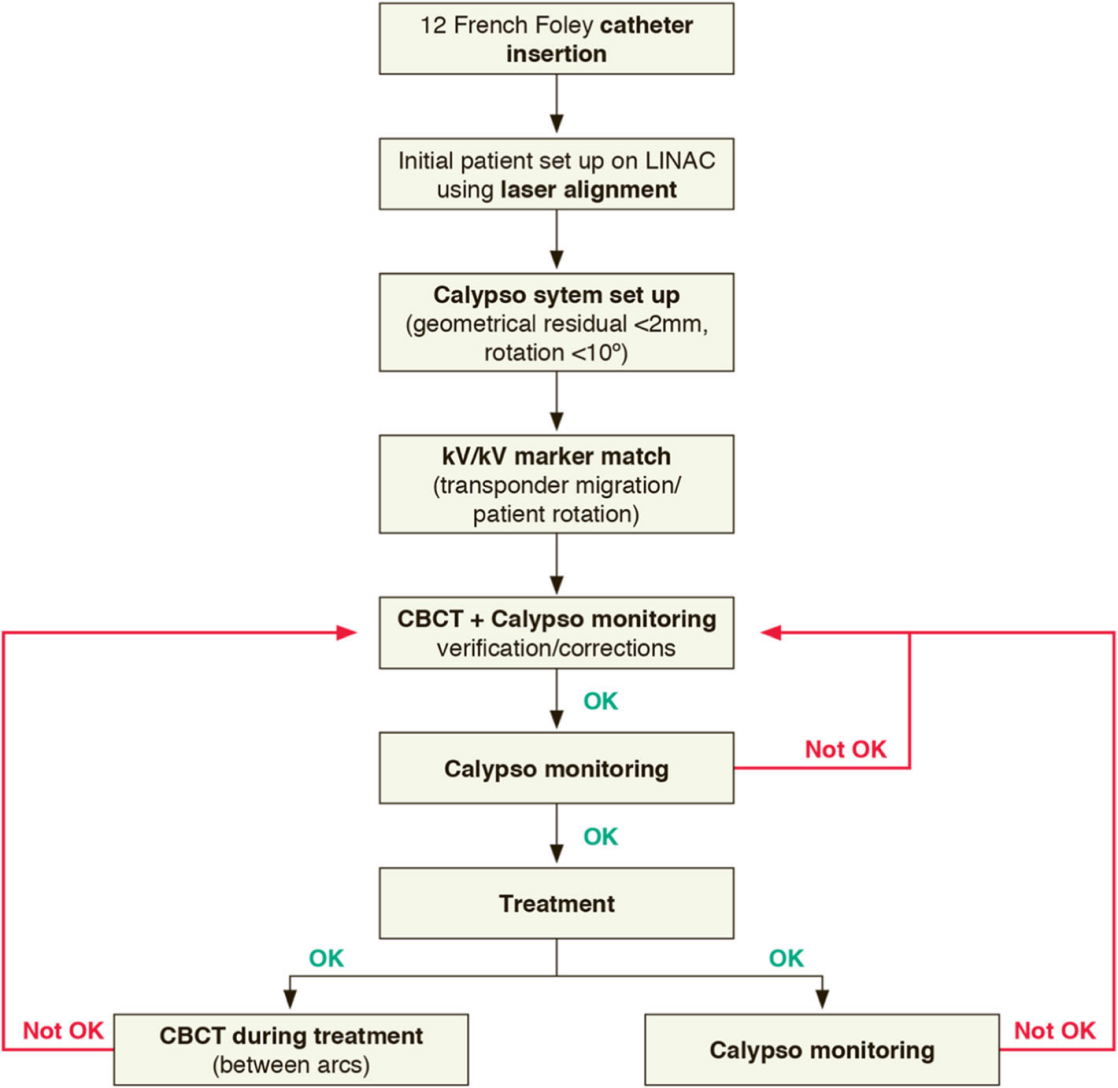
Image-guided flowchart as per protocol

As volumetric modulated arc therapy (VMAT) techniques are mandatory in this trial, the definition of volumes and the dose reporting shall be in accordance with the ICRU (*International Commission on Radiation Units and Measurements*) report 83. The dose variation in the PTV shall be assessed by the near-minimum dose (D98%) and near-maximum dose (D2%). The proposed treatment verification schedule is presented in Fig. 1 using a threshold limit for *Calypso*® of ±3 mm with geometric check limits set to 2 mm (geometrical residual) and rotations of 10° (default values). Table 1 summarizes the dose constraints for the OAR [24].

**Table 1:** Dose constraints

Structure	Standardized nomenclature	Dosimetric Parameter	Per Protocol	Acceptable Variation
PTV_19	PTV_19	D98% (Dnear min)	≥ 95% (i.e. 18 Gy)	
		D2% (Dnear max)	≤ 107% (ie. 20.3Gy)	D5% ≤ 107%, D2% ≤ 110%
Rectal_wall	Rectal_wall	V9.5Gy (50%)	< 40%	40–50%
		V15Gy (80%)	< 15%	15–20%
		V17Gy (90%) (NTD 68 Gy)	< 10%	< 15%
		V19Gy (100%) (NTD 83.6 Gy)	< 5%	< 10%
		V20Gy (105%) (NTD 92 Gy)	< 1 cc	
Bladder_wall	Bladder_wall	V9.5Gy (50%)	< 50%	50–60%
		V15Gy (80%)	< 20%	20–30%
		V17Gy (90%) (NTD 68 Gy)	< 15%	15–20%
		V19Gy (100%) (NTD 83.6 Gy)	< 10%	< 15%
		V20Gy (105%) (NTD 92 Gy)	< 1 cc	
Femurs (each)	Femur_Rt Femur_Lt	V14Gy (NTD 48 Gy)	< 5%	5–10%
Penile bulb	Penile_bulb	V14Gy (NTD 48 Gy)	< 5%	5–10%
Urethra PRV	Urethra_PRV	D98% (Dnear min)	≥ 95% (i.e. 16.2 Gy)	
		D5%	≤ 107% (i.e. 18.2 Gy)	
		D2% (Dnear max)	≤ 110% (i.e. 18.7 Gy)	

##### Radiation Therapy Quality Assurance (RTQA)

A RTQA program is based in Geneva and is an important asset of this multicentric trial. Electronic submission and assessment of target and OaR volumes, in addition to treatment plans as well, is a major step before any treatment can be validated and approved.

#### Follow-up

Patients are seen at day-5 after SBRT, at weeks 6 and 12, every 6 months for 2 years post treatment, and yearly up to 5 years of follow-up. A clinical update and a physical exam are conducted by recording any acute and/or late AE and the sequential PSA measurements. IPSS, IIEF-5, and QoL (EPIC-26 questionnaire) assessments are also performed. Radiological investigations including mpMRI, bone scan, choline-PET, and/or PSMA-PET will be repeated in case of progression, either biochemical or clinical.

#### Statistical analysis

##### Sample size

ᅟ

### Phase I, safety evaluation

Only acute GU and/or GI grade 3 or higher toxicity will be considered for the safety evaluation. Three patients will be treated and evaluated for toxicity at 3 months. If the first 3 patients undergo any grade ≥ 3 toxicity or if 2 patients under go grade 4 toxicity, the trial will stop.

Otherwise, 3 additional patients will be recruited and treated. If 2/6 patients or fewer present with grade 3 AE, the trial will go on phase II and include 39 additional patients. Regarding these 6 patients, if one or more present with grade 4 toxicity, a meeting of the *Data Safety and Monitoring Board* (DSMB) will be carried on.

### Phase II, efficacy evaluation

Based on published SBRT results on 5 fraction-trials and on one series of HDR-BT with 19 Gy single shot, a 3-yearsbRFS of 96% may be expected. Assuming this hypothesis and an expected dropout rate of 10%, 45 patients (including the 6 included in the phase I study) will allow evaluating 3-year bRFS with a 97.5% lower one-sided confidence interval and a width of the interval of 0.06 (upper bound = 90%).

### Data analysis

Data description will be performed using the mean, standard deviation, median, and range for quantitative variables and the percentage for qualitative ones.

#### Phase I

Safety analyses shall be performed on the safety-evaluable population, defined as all subjects treated with the single shot SBRT. Toxicity and grade will be described at each follow-up visit according to the specificity of every assessed endpoint, including the number and fraction of patients with at least one AE.

#### Phase II- statistical analysis of the primary endpoint

Efficacy analyses will be performed on an intention to treat basis (i.e., involving those patients following the major inclusion criteria and repeated one per protocol set). The primary endpoint is 3-years bRFS. bRFS, and its 97.5% one-sided confidence interval (CI), will be determined using the *Kaplan Meier* method. If the expected value of 96% isn’t included in this interval, the efficacy of the experimental treatment will be questioned.

#### Phase II - statistical analysis of the secondary endpoints

Toxicity analyses will be performed. As for acute toxicity, late AE and grading will be reported at each follow up visit. Median survival time and its 95% bilateral confidence intervals (PFS, clinical PFS, local PFS, OS, and PCSS) will be determined using the *Kaplan Meier* method. The rate of survival will be given at 1, 2, 3, and 5 years. Univariate and multivariate *Cox* regression models will be fit to assess the effect of relevant baseline clinical and pathologic features on bRFS, PFS, and OS. Hazard ratio (HR) will be given with their 95% confidence intervals. Median follow-up will be estimated using the reverse *Kaplan Meier* method. QoL scores (EPIC, IPSS, IIEF-5) will be described at each clinical surveillance follow-up time by the mean, standard deviation, median, and range. Domain scores will be calculated only if sufficient items are completed in accordance with the relevant scoring manual. Mixt models will be used to characterize changes in QoL across time taken into account for confounding clinical and pathologic factors. All statistical analysis will be performed with SAS 9.4. *p*-values less than 5% will be considered as significant.

## Discussion

Optimal management of localized low- or intermediate risk prostate cancer remains a challenging situation, with different treatment options available ranging from active surveillance to radical treatment [25]. Considering that patients under active surveillance have a greater risk of disease progression and metastases compared to definitive treatments [26], the choice of the more appropriate curative option is a crucial issue for clinicians.

SBRT represents a valid treatment option for these patients, combing a good toxicity profile, promising disease control rates, and limited impact on QoL. Recently reported, preliminary results of the Scandinavian phase III HYPO-RT-PC trial found in 1200 intermediate-risk prostate cancer patients similar biochemical control rates and toxicities results with extreme hypofractionation (42.7 Gy in seven fractions of 6.1 Gy over two and a half weeks) compared to standard fractionation 78 Gy [27]. However, the optimal SBRT fractionation schedule for curative treatment of patients with localized prostate cancer is, so far, unknown. Based on the promising results of HDR-BT monotherapy treatments [15, 28, 29], single-shot SBRT may represent an appealing treatment modality for prostate cancer patients.

To our knowledge, ONE SHOT is the first phase I/II trial assessing the efficacy and safety of a single-dose SBRT treatment for patients with localized prostate cancer in a multicenter setting. ONE SHOT proposes the same treatment technique under a strict RTQA protocol for all patients. By reducing significantly the beam-on time [30], our single shot linac-based FFF VMAT technique with intra-fractional control motion is expected to guarantee not only treatment reproducibility [31] but also minimize intra-fraction dose uncertainties [32]. Moreover, the implementation of patient-reported outcome using validated questionnaires will provide a reliable evaluation of the clinical impact of a single-dose SBRT by reporting a longitudinal follow-up on the long-term of radiation-induced side effects compared to physician-reported data.

While waiting for results of presently ongoing randomized phase III trials comparing SBRT with other fractionation or treatment, results of the ONE SHOT trial, if positive, may help to design subsequent studies exploring the role of SBRT monotherapy in the exclusive RT treatment of localized disease.

## Abbreviations

AE: Adverse event
ASTRO: American Society for Radiation Oncology
bRFS: Biochemical relapse-free survival
CTV: Clinical target volume
DSMB: Data safety and monitoring board
EPIC 26: Expanded prostate cancer index composite
FFF: Flattening filter-free
HDR-BT: High-dose rate brachytherapy
ICRU: International Commission on Radiation Units and Measurements
IIEF-5: International Index of Erectile Function
IPSS: International Prostate Symptoms Score
LQ: Linear quadratic
mpMRI: Multi-parametric Magnetic Resonance Imaging
NCI CTCAE: National Cancer Institute Common Terminology Criteria for Adverse Events
OAR: Organs at risk
OS: Overall survival
PCSS: Prostate cancer-specific survival
PFS: Progression-free survival
PRV: Planning risk volume
PSA: Prostate specific antigen
PTV: Planning target volume
QoL: Quality of life
RT: Radiotherapy
RTOG: Radiation Therapy Oncology Group
RTQA: Radiation Therapy Quality Assessment
SBRT: Stereotactic body radiotherapy
SV: Seminal vesicles
VMAT: Volumetric modulated arc therapy
WHO: World Health Organization
