# Correction to: ONE SHOT - single shot radiotherapy for localized prostate cancer: study protocol of a single arm, multicenter phase I/II trial

**DOI:** 10.1186/s13014-018-1131-x

**Published:** 2018-09-19

**Authors:** Thomas Zilli, Marta Scorsetti, Daniel Zwahlen, Ciro Franzese, Robert Förster, Niccolò Giaj-Levra, Nikolaos Koutsouvelis, Aurelie Bertaut, Michel Zimmermann, Giuseppe Roberto D’Agostino, Filippo Alongi, Matthias Guckenberger, Raymond Miralbell

**Affiliations:** 1 Radiation Oncology, Geneva University Hospital, CH-1211, 14 Geneva, Switzerland; 2 Faculty of Medicine, Geneva University, Geneva, Switzerland; 3 Radiation Oncology, Humanitas University, Rozzano, Milan, Italy; 4 Radiation Oncology, Humanitas Research Hospital and Cancer Center, Rozzano, Milan, Italy; 5 Radiation Oncology, Kantonsspital Graubünden, Chur, Switzerland; 6 Radiation Oncology, University Hospital Zürich, Zürich, Switzerland; 7 Radiation Oncology, Sacro Cuore Don-Calabria, Negrar, Italy; 8 Methodology and biostatistic unit, Centre Georges François Leclerc, Dijon, France; 9 Faculty of Medecine, University of Brescia, Brescia, Italy

## Correction

Following publication of the original article [1], the authors reported that one of the authors’ names is spelled incorrectly. In this Correction the incorrect and correct author name are shown. The original publication of this article has been corrected.

Originally the author name has been published as:

- Nikolaos Koustouvelis

The correct author name is:

- Nikolaos Koutsouvelis
